# Vitamin D supplementation and mortality among critically ill adults: a systematic review and meta-analysis

**DOI:** 10.3389/fnut.2026.1796272

**Published:** 2026-06-22

**Authors:** Chienhsiu Huang

**Affiliations:** Department of Internal Medicine, Dalin Tzu Chi Hospital, Buddhist Tzu Chi Medical Foundation, Chiayi, Taiwan

**Keywords:** 25-hydroxyvitamin D level, critically ill adults, delivery route, mortality rate, supplementation dosage, vitamin D

## Abstract

**Background:**

Randomized controlled trials have failed to demonstrate the effects of vitamin D supplementation on reducing the mortality rate in critically ill adults. A *post hoc* study revealed that vitamin D administration was linked to a lower 28-day mortality rate. The purpose of this meta-analysis was to investigate how vitamin D supplementation affects mortality in critically ill adults. The effects of vitamin D supplementation, such as baseline 25-hydroxyvitamin D level, vitamin D supplementation dosage, and vitamin D delivery route, were given special attention in relevant subgroups.

**Methods:**

The inclusion criteria for eligible studies were as follows: (1) randomized controlled trials; (2) critically ill adults; (3) the intervention group was given vitamin D or a vitamin D metabolite without any restrictions on type, dosage, duration, or route of administration; and (4). the outcome of mortality in critically ill adults.

**Results:**

Subgroup analysis revealed the following findings: (1) there was a trend toward reduced mortality in critically ill adults whose baseline 25-hydroxyvitamin D level was less than 20 ng/mL (RR = 0.87, *p* = 0.05, 95% CI = 0.76–1.00, *I*^2^ = 31%). Meta-analysis revealed a relative risk of mortality of 0.87, indicating that patients with baseline 25-hydroxyvitamin D level less than 20 ng/mL receiving vitamin D supplementation had a 13% lower risk of death than controls; (2) mortality was significantly reduced in patients who received ≤300,000 IU of vitamin D compared with those who received the placebo, (RR = 0.56, *p* < 0.0001, 95% CI = 0.42–0.74, *I*^2^ = 0%); (3) administration by intramuscular injection or intravenous injection significantly reduced the risk of mortality (RR = 0.59, *p* = 0.0006, 95% CI = 0.44–0.80, *I*^2^ = 0%).

**Conclusion:**

This meta-analysis revealed that vitamin D supplementation significantly reduced mortality in critically ill adult patients, but not all patients benefited. The benefit was observed in three subgroups of patients as below: (1) critically ill adults with baseline 25-hydroxyvitamin D levels below 20 ng/mL; (2) critically ill adults receiving ≤300,000 IU of vitamin D supplementation; (3) critically ill adults receiving the supplement via intramuscular or intravenous injection.

**Systematic review registration:**

CRD420251152462.

## 1 Introduction

Vitamin D, which is a fat-soluble vitamin, regulates the amounts of calcium and phosphorus in bone metabolism. Vitamin D is used in clinical practice to treat osteoporosis, hyperparathyroidism, and hyperproliferative skin diseases. There are additional non-skeletal pleiotropic effects of vitamin D. These effects, which include cardiovascular modulation and the immune system’s reaction to acute inflammation and infection, may be crucial for recovery from critical illness (1–4). Numerous investigations have shown that vitamin D insufficiency is widespread in critically ill adults. Moreover, 78.1% of adult septic shock patients in Chae et al.’s (5) study (lower vitamin D levels than <20 ng/mL), 69.1% of ICU (intensive care unit) patients in Sistanian et al.’s (6) study (lower vitamin D levels than 20 ng/mL), 80.4% of critically ill adults in Azim et al.’s (7) study (lower vitamin D levels than 60 nmoL/L), and 93.5% of ICU patients in Vosoughi et al.’s (8) study had low baseline 25-hydroxyvitamin D levels (lower vitamin D levels than 30 ng/ mL). In the study of Higgins et al. (9) showed that of analyzable patients, 50 (26%) were deficient (≤30 nmol/L) and 109 (56%) were insufficient (>30 and ≤60 nmol/L). Baseline 25(OH) D levels decreased significantly in all patients after 3 days in the ICU and remained significantly lower through 10 days. In the study of Anwar et al. (10) showed that median level of vitamin D was 6.4 ng/mL among patients with a prolonged stay and 8.08 ng/mL among patients with a short stay. Over the course of the ICU stay, these levels remained notably lower. Numerous studies have shown that vitamin D insufficiency is linked to increased incidence of infection, sepsis, acute respiratory failure and acute kidney injury in critically ill adults (11–16). According to the 2019 ESPEN recommendations on clinical nutrition in the ICU, a high dosage of vitamin D3 (500,000 IU) as a single dose can be given within a week of admission to critically ill patients with low plasma levels (25-hydroxyvitamin D concentration < 12.5 ng/mL) (17). The 2023 clinical nutrition guidelines updated the uncertainty around the timing and dosage of vitamin D administration in critically ill patients (18). Two large randomized controlled trials (RCTs) (VITdAL-ICU and VIOLET) failed to demonstrate the effects of vitamin D supplementation on reducing mortality in critically ill adults (19, 20). However, a *post hoc* analysis of the VITdAL-ICU study in which participants who died or were discharged within 7 days (patients who were too ill or too healthy) were excluded revealed that vitamin D administration was linked to a lower 28-day mortality rate. A survival benefit was linked to an increase in 25-hydroxyvitamin D levels on day three (21). A narrative review by Wang et al. (22) revealed that vitamin D supplementation is safe and that its effect on overall mortality remains uncertain. However, the therapeutic importance of vitamin D supplementation in critically ill adults remains unclear. In our opinion, vitamin D supplementation in critically ill adults cannot reduce mortality in every type of patient. Subgroups of critically ill adults whose mortality is reduced from vitamin D supplementation should be identified. The purpose of this meta-analysis was to investigate how vitamin D supplementation affects mortality in critically ill adults. We further aimed to verify which subgroups of patients who received vitamin D supplementation significantly benefited from a reduction in mortality in the current meta-analysis. The effects of vitamin D supplementation on relevant subgroups, such as baseline 25-hydroxyvitamin D levels, vitamin D supplementation dosage, and vitamin D supplementation delivery route (including oral, enteral, intravenous, and intramuscular), were given special attention.

## 2 Methods

### 2.1 Data search strategy

Our analysis followed the Preferred Reporting Items for Systematic Reviews and Meta-Analyses guidelines. From January 1, 1990, to June 30, 2025, the following search terms were used to search the PubMed, Web of Science, and Cochrane Library databases: (vitamin D OR 25 hydroxy vitamin D OR 25(OH) D OR calcitriol OR cholecalciferol OR ergocalciferol) AND (critically ill OR critical care) AND (intensive care OR ICU).

### 2.2 Selection criteria

The predefined criteria for eligible studies were as follows: (1) RCTs; (2) critically ill adults (age > 18 years, with no upper age limit); (3) the control group received a placebo or no medication, whereas the intervention group received vitamin D or a vitamin D metabolite without any restrictions on its type, dosage, duration, or route of administration(including oral, enteral, intravenous, and intramuscular); and (4) the primary outcome was mortality in critically ill adults. We first evaluated 90-day mortality, followed by 28-day mortality and hospital mortality. We excluded studies that reported only ICU mortality. We excluded case reports, conference abstracts, reviews, comments, and *post hoc* analyses. Upon discovering study with the same patient population, only the most current update was included. Articles published in all languages were included.

### 2.3 Data extraction

Data concerning study characteristics (year of publication, study design, and study country), baseline 25-hydroxyvitamin D level, form of vitamin D or a vitamin D metabolite, type of intervention (route and dosage of vitamin D) and mortality (90-day mortality, 28-day mortality, and hospital mortality) were extracted.

### 2.4 Definitions

According to the widely used definition, vitamin D status is categorized as follows: Normal vitamin D was defined as a serum 25-hydroxyvitamin D level > 30 ng/mL. Insufficient vitamin D was defined as a serum 25-hydroxyvitamin D level of 20–30 ng/mL. Vitamin D deficiency was defined as a serum 25-hydroxyvitamin D level < 20 ng/mL. Severe vitamin D deficiency was defined as a serum 25-hydroxyvitamin D level < 12 ng/mL. The systematic review conducted by Kearns et al. concluded that administration of single doses of vitamin D3 equal to or exceeding 300,000 IU is most efficacious in enhancing vitamin D status. However, reduced dosages may prove adequate for specific demographic groups. It is imperative that vitamin D dosages surpassing 500,000 IU be administered with caution to mitigate potential adverse effects (23). According to the study of Kearns et al., doses of >300,000 IU and ≤300,000 IU of vitamin D were defined as high and low doses, respectively. We performed subgroup analyses to assess the route of supplementation, dosage of vitamin D, and baseline 25-hydroxyvitamin D level to explore which subgroups of patients could exhibit a reduction in mortality.

### 2.5 Risk of bias assessment and statistical analysis

We assessed the risk of bias in each study using the Cochrane Risk-of-Bias Tool 2.0 for RCTs. Two reviewers (SuFangGuo and LiChen Lin) examined publications independently to avoid bias. When disagreement occurred, a third author (Tiju Tang) resolved the issue. Data were entered into the Cochrane Review Manager software RevMan 5.4. Differences were expressed as risk ratios (RRs) with 95% confidence intervals (CIs) for dichotomous outcomes. The significance of the pooled ratios was determined by the *Z* test, and a *p* value less than 0.05 was considered statistically significant. Cochran’s *Q* and *I*^2^ tests were used to assess the heterogeneity of the data included in each outcome. A significance level of *p* < 0.1 was considered for the *Q* statistic, and *I*^2^ values above 50% were considered evidence of significant heterogeneity to account for the limited number of studies included in many of the analyzed outcomes. The fixed-effects model was used when the effects were assumed to be homogenous, while the random-effects model was used when they were heterogeneous.

## 3 Results

### 3.1 Databases, study selection, and characteristics of included studies

**Figure 1:**
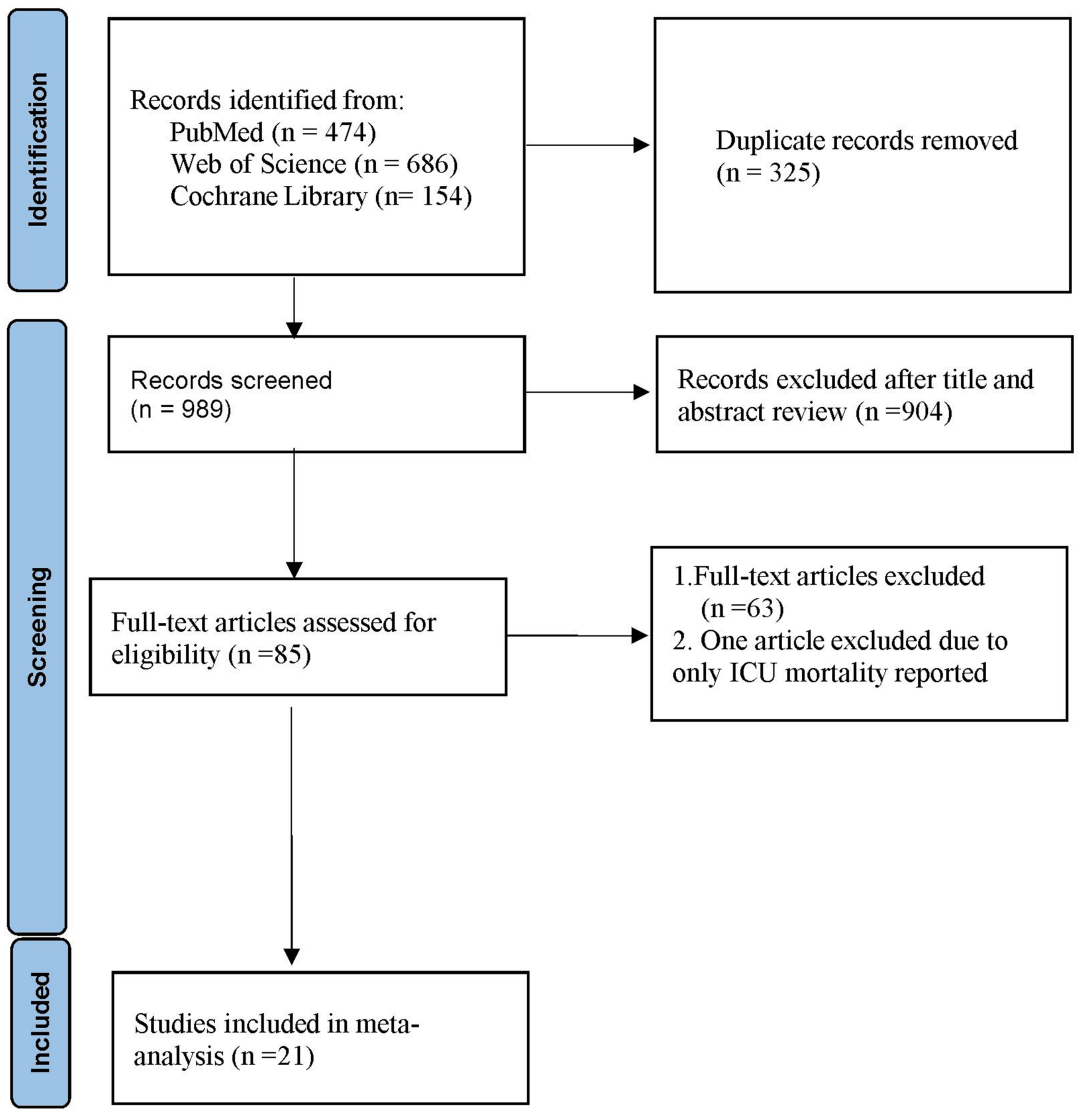
Flow diagram of the study selection process.

**Figure 2:**
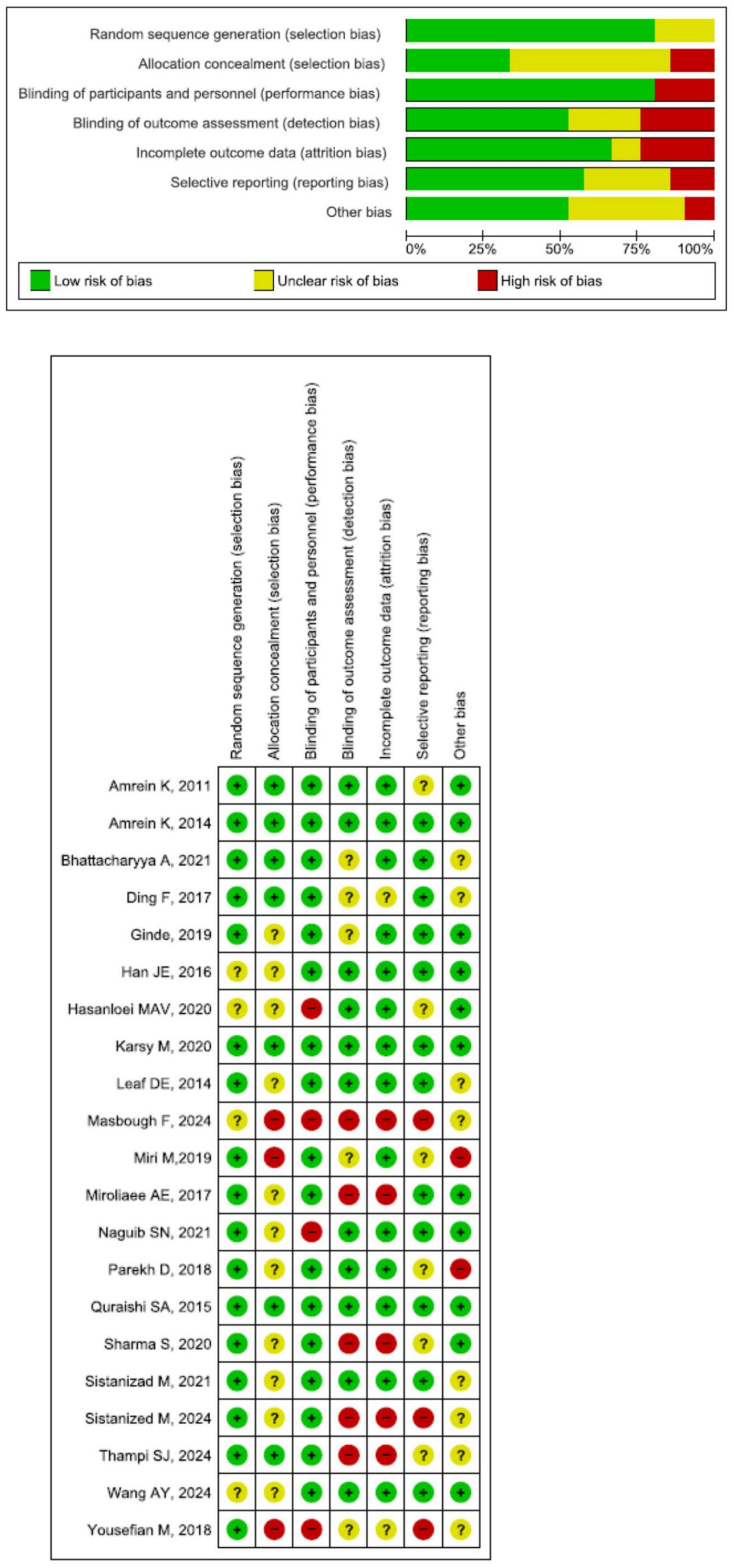
Risk of bias of the 21 included studies.

The details of the study selection process are shown in Figure 1. A total of 474, 686 and 154 studies were identified from the initial search results from PubMed, Web of Science and the Cochrane Library, respectively. There were 325 duplicate articles. A total of 904 irrelevant studies were identified by reading the title and abstract. After excluding duplicates and irrelevant studies, 85 potentially relevant articles remained. After the full-text article review, 63 articles were excluded because they lacked results comparing the mortality of critically ill adults who received vitamin D supplements with those who received placebo supplements. One study was excluded because it only reported ICU mortality (24). Finally, 21 studies were included in the meta-analysis (19, 20, 25–43). The main characteristics and risk of bias of the 21 included studies are shown in Table 1 and Figure 2.

**Table 1:** Characteristics of the included studies.

Author/year	Country	Baseline 25(OH) D level (ng/ml) (Vit. D group/placebo)	Route of Vit. D	Dosage of Vit. D	No of patients (Vit. D group/placebo)
Amrein et al./2011 (25)	Austria	13.1/14.1	Oral	540,000 IU	237/238
Amrein et al./2014 (19)	Austria	13.0/13.1	Oral/enteral	540,000 IU & 90,000 IU x5	12/13
Leaf et al./2014 (26)	USA	No data	IV	Calcitriol 2ug	36/31
Quraishi et al./2015 (27)	USA	A.15.0 and B.17.0/19.0	Oral	A.200000 IU B.400000 IU	20/10
Han et al./2016 (28)	USA	A.1.23.2 and B.20.0/21.5	Oral	A.50000 IU B.100000 IU X 5	20/10
Miroliaee et al./2017 (29)	Iran	19.5/17.12	IM	300,000 IU	24/22
Ding et al./2017 (30)	China	3.92/3.92	IM	300,000 IU	29/28
Parekh et al./2018 (31)	UK	18.96/18.52	Oral	300,000 IU	33/35
Yousefian et al./2018 (32)	Iran	8.85/10.52	IM	300,000 IU x 3	33/33
Ginde et al./2019 (20)	USA	11.2/11.0	Oral	540,000 IU	531/528
Miri et al./2019 (33)	Iran	8.43/11.35	IM	300,000 IU	22/18
Karsy et al./2020 (34)	USA	14.6/13.9	Oral	540,000 IU	134/133
Sharma et al./2020 (35)	India	18.30/15.15	Oral	120,000 IU	20/15
Hasanloei et al./2020 (36)	Iran	A.1.6.83B.7.46/6.8	Oral/IM	A.50000 IU X 6 B.300000 IU	48/24
Sistanizad et al./2021 (37)	Iran	7.3/5.24	IM	300,000 IU	16/14
Bhattacharyya et al./2021 (38)	India	12.05/15.47	Oral	540,000 IU	63/63
Naguib et al./2021 (39)	Egypt	21.0/19.1	Oral	Alfacalcidol 2ug/day	45/42
Sistanizad et al./2024 (40)	Iran	11.37/12.27	IV	Calcitriol 1 ug X 3	14/13
Thampi et al./2024 (41)	UK	No data	IM	Calcitriol (300,000 IU)	76/76
Wang et al./2024 (42)	Taiwan	14.4/13.1	Enteral	569,600 IU	41/20
Masbough et al./2024 (43)	Iran	15.95/17.84	IM	300,000 IU	19/16

USA, United States of America; UK, United Kingdom; Vit. D, Vitamin D; IV, Intravenous injection; IM, Intramuscular injection; IU, international unit; ug, Microgram; ng/mL.

### 3.2 Mortality in critically ill adults

**Figure 3:**
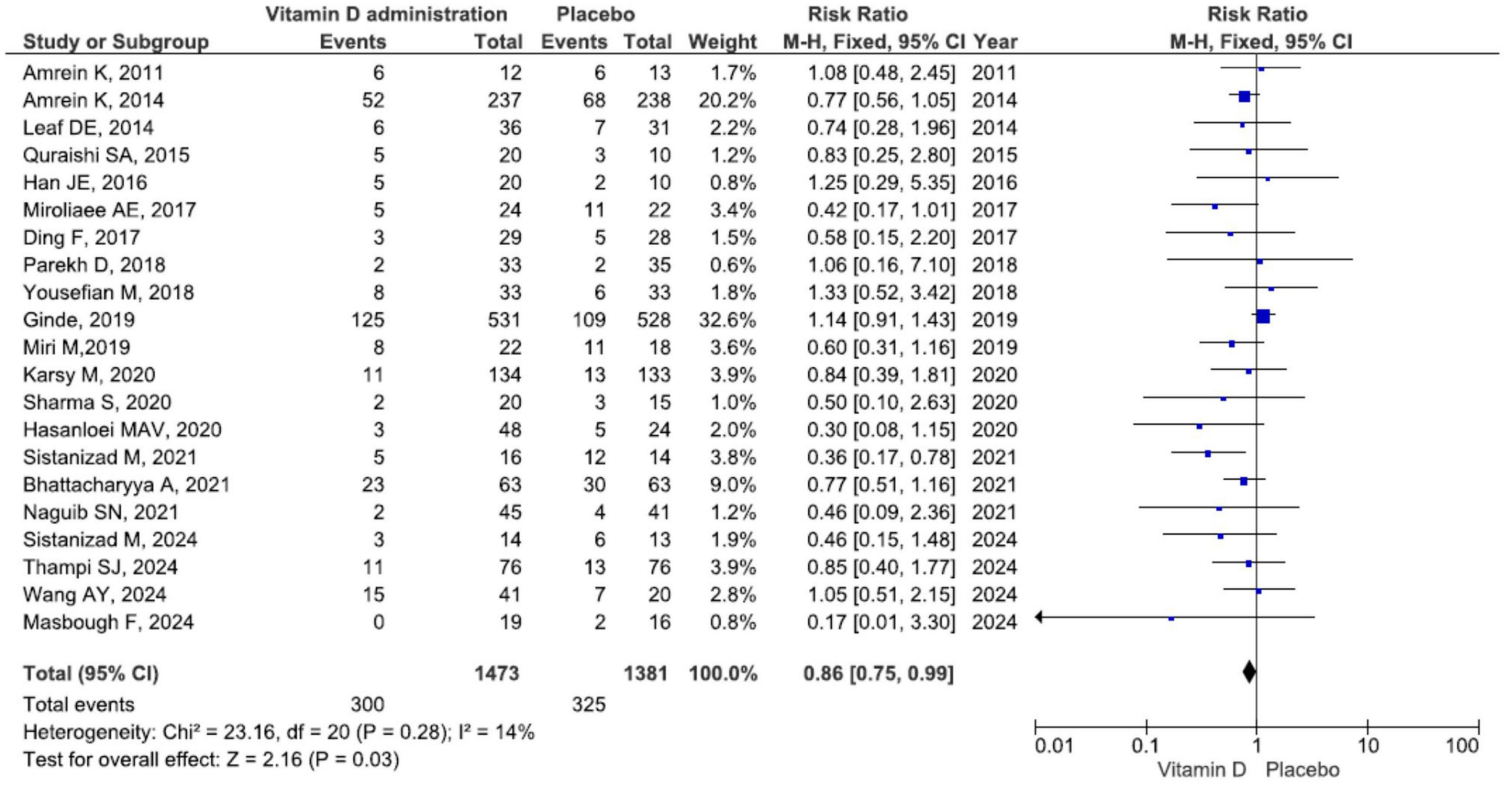
Mortality in critically ill patients: vitamin D compared to placebo.

Five studies provided 90-day mortality (20, 28, 31, 38, 42), 11 studies reported 28-day mortality (19, 26, 27, 29, 30, 33, 34, 37, 40, 41, 43), and five studies reported hospital mortality (25, 32, 35, 36, 39) in the current meta-analysis. Compared with the placebo, vitamin D supplementation significantly reduced mortality (*p* = 0.03, RR = 0.86, 95% CI = 0.75–0.99, *I*^2^ = 14%) (Figure 3). After the VIOLET study was removed, critically ill adults who received vitamin D supplementation experienced a significant reduction in mortality (*p* = 0.0002, RR = 0.73).

### 3.3 High-dose vitamin D versus low-dose vitamin D subgroups

**Figure 4:**
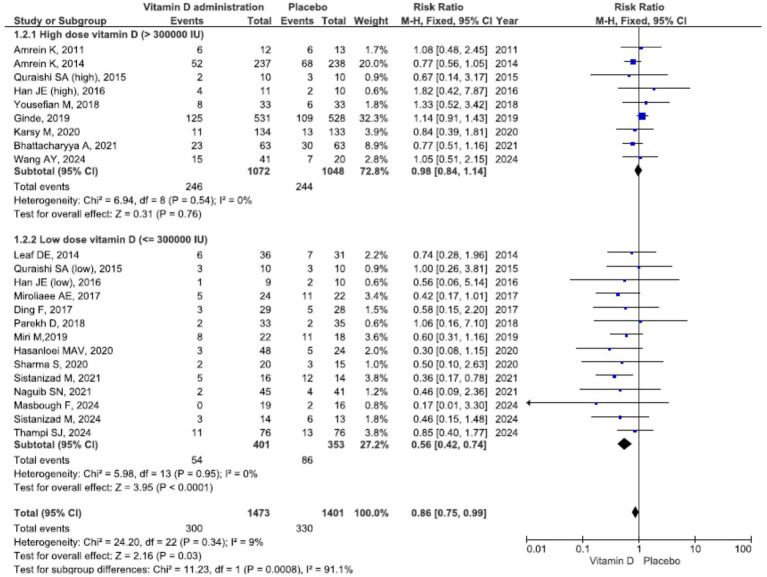
Subgroup analysis of administration dosage for mortality in critically ill patients: vitamin D compared to placebo.

Different dosages of vitamin D were administered, and the results showed that mortality was significantly reduced in patients who received low-dose vitamin D compared with those who received the placebo, (*p* < 0.0001, RR = 0.56, 95% CI = 0.42–0.74, *I*^2^ = 0%) Meta-analysis revealed a relative risk of mortality of 0.56, indicating that patients who received ≤300,000 IU of vitamin D supplementation had a 44% lower risk of death than controls (26–31, 33, 35–37, 39–41, 43). Compared with placebo, high-dose vitamin D did not significantly reduce mortality (*p* = 0.76, RR = 0.98, 95% CI = 0.84–1.14, *I*^2^ = 0%) (19, 20, 25, 27, 28, 32, 34, 38, 42) (Figure 4). After the VIOLET study was removed, compared with placebo, high-dose vitamin D did not significantly reduce mortality (*p* = 0.12, RR = 0.85).

### 3.4 Intramuscular (IM)/intravenous (IV) versus enteral/oral subgroups

**Figure 5:**
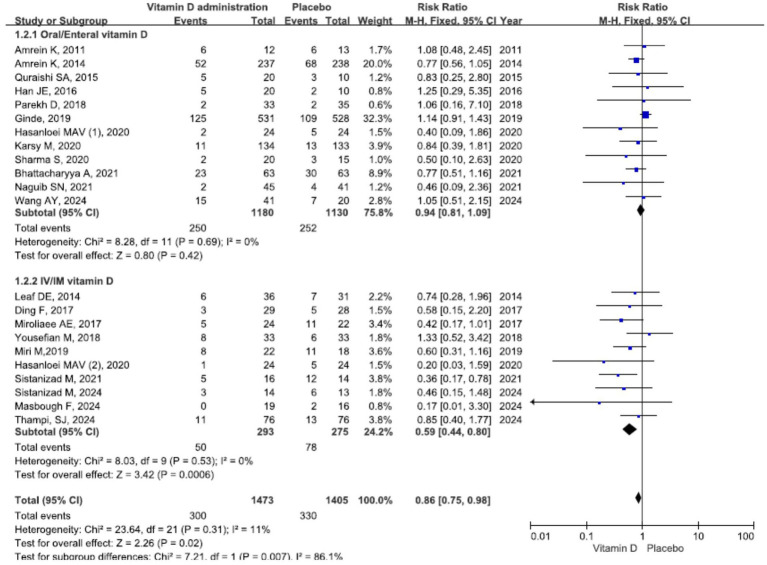
Subgroup analysis of administration route for mortality in critically ill patients: vitamin D compared to placebo.

Studies using IV/IM as a route of administration revealed significantly lower mortality compared with the placebo group (*p* = 0.0006, RR = 0.59, 95% CI = 0.44–0.80, *I*^2^ = 0%) Meta-analysis revealed a relative risk of mortality of 0.59, indicating that patients who received intramuscular injection or intravenous injection vitamin D supplementation had a 41% lower risk of death than controls (19, 20, 25, 27, 28, 31, 34–36, 38, 39, 42). Conversely, compared with placebo, enteral/oral vitamin D administration did not significantly reduce mortality (*p* = 0.42, RR = 0.94, 95% CI = 0.81–1.09, *I*^2^ = 0%) (26, 29, 30, 32, 33, 36, 37, 40, 41, 43) (Figure 5). After the VIOLET study was removed, critically ill adults who received enteral/oral vitamin D supplementation experienced a significant reduction in mortality (*p* = 0.03, RR = 0.79).

### 3.5 Baseline 25-hydroxy vitamin D level

**Figure 6:**
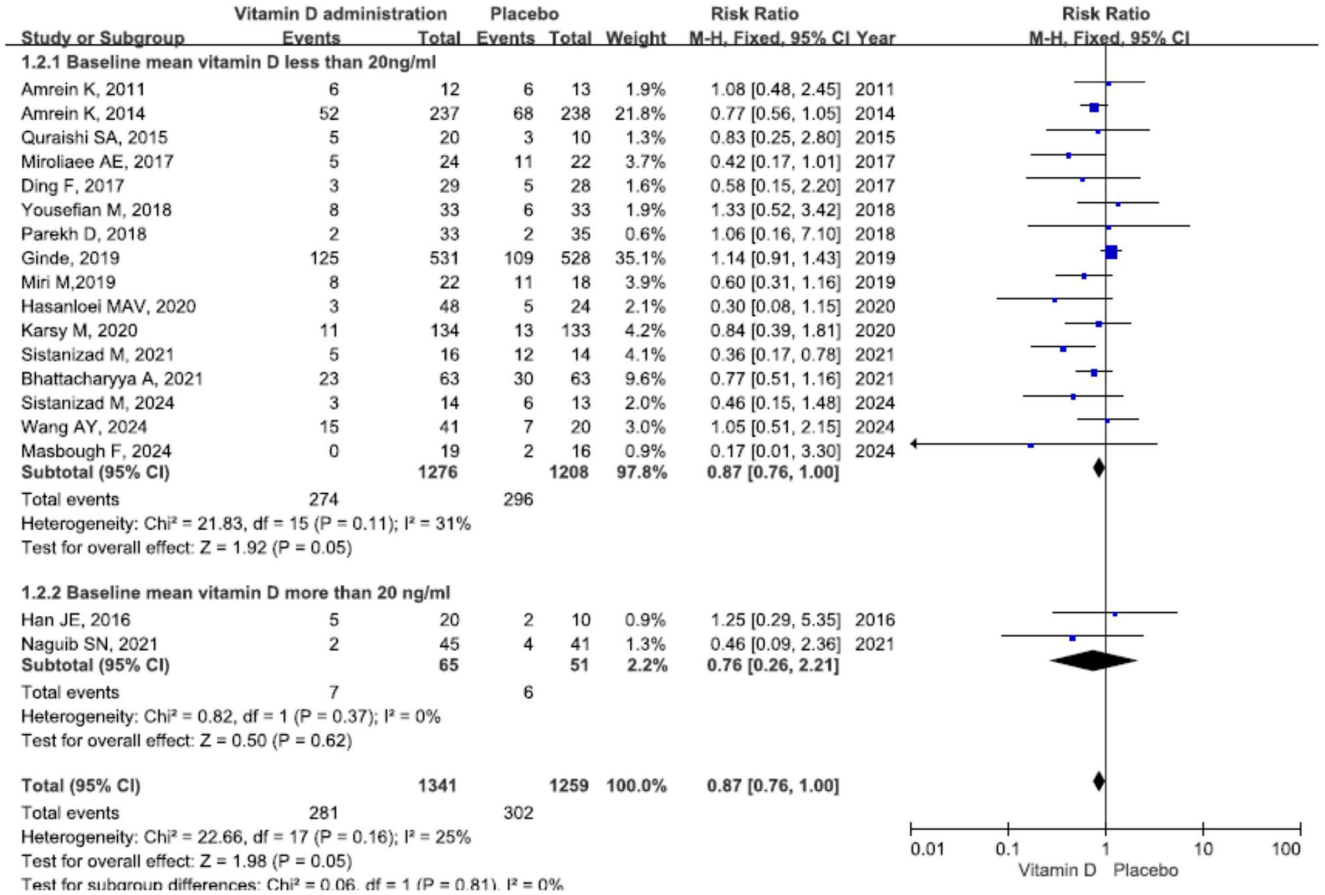
Subgroup analysis of baseline mean vitamin D level for mortality in critically ill patients: vitamin D compared to placebo.

**Figure 7:**
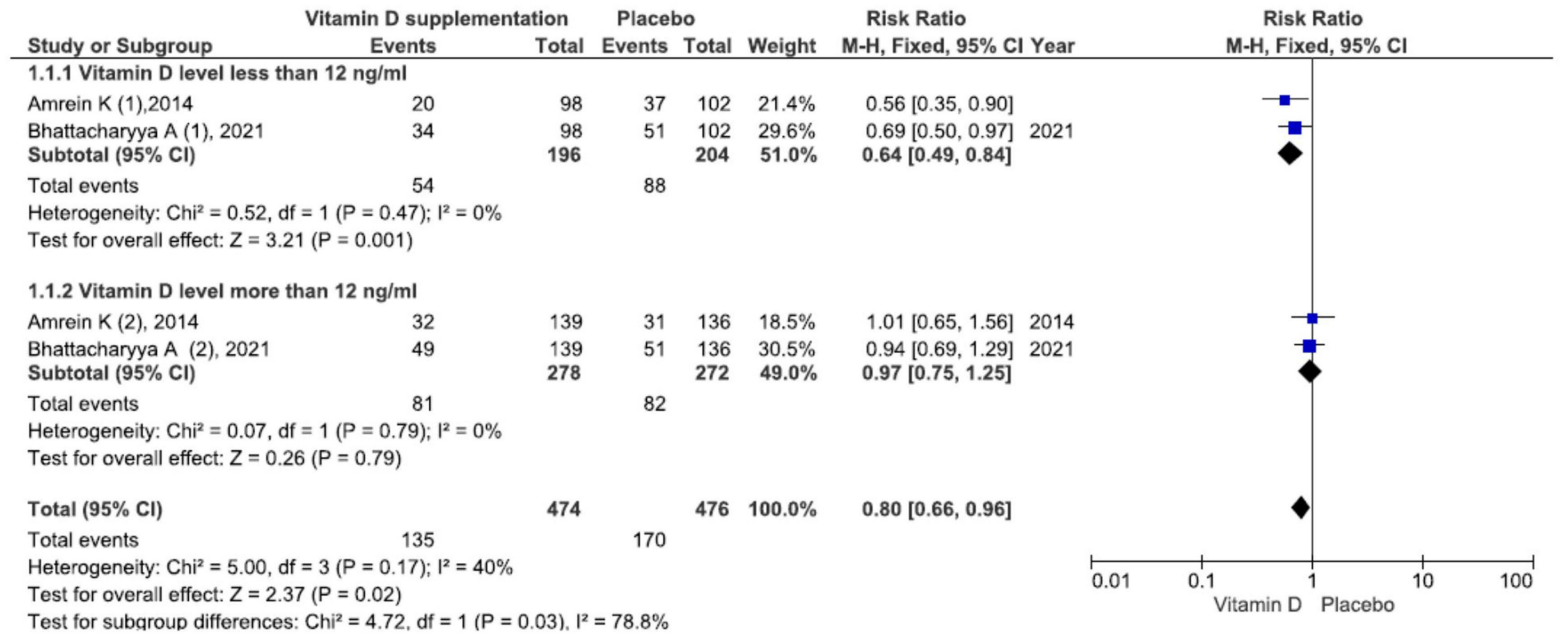
Subgroup analysis of baseline mean vitamin D level less than 12 ng/mL for mortality in critically ill patients: vitamin D compared to placebo.

Baseline 25-hydroxyvitamin D levels were analyzed, and mortality tended to decrease in patients whose baseline 25-hydroxyvitamin D level was less than 20 ng/mL compared with that in patients who received placebo (*p* = 0.05, RR = 0.87, 95% CI = 0.76–1.00, *I*^2^ = 31%) Meta-analysis revealed a relative risk of mortality of 0.87, indicating that patients with baseline 25-hydroxyvitamin D level less than 20 ng/mL receiving vitamin D supplementation had a 13% lower risk of death than controls (19, 20, 25, 27, 29–34, 36–38, 40, 42, 43). After the VIOLET study was removed, mortality tended to decrease in patients whose baseline 25-hydroxyvitamin D level was less than 20 ng/mL compared with that in patients who received placebo (*p* = 0.0004, RR = 0.72). In the subgroup of patients whose baseline 25-hydroxyvitamin D level was more than 20 ng/mL, there was no significant reduction in mortality compared with placebo (*p* = 0.62, RR = 0.76, 95% CI = 0.26–2.21, *I*^2^ = 0%) (28, 39) (Figure 6). Two studies provided baseline 25-hydroxyvitamin D levels <12 ng/mL, and mortality was significantly lower in patients whose baseline 25-hydroxyvitamin D level was less than 12 ng/mL compared with those who received a placebo (*p* = 0.001, RR = 0.64, 95% CI = 0.49–0.84, *I*^2^ = 0%) (19, 38) (Figure 7).

## 4 Discussion

There is awareness of the significance of vitamin D beyond the preservation of musculoskeletal well-being. The correlation of chronic illness and vitamin D insufficiency has engendered scholarly interest into the function of vitamin D in critical illness. Serum concentrations of vitamin D metabolites and their binding proteins are diminished in critically ill individuals; the mechanisms are complex (44). Vitamin D modulates both the innate and adaptive immune systems. Vitamin D insufficiency results in immune dysregulation and has been posited as a fundamental mechanism of a spectrum of infectious and autoimmune disorders (45). Markers of systemic inflammation typically increase among vitamin D deficient critically ill patients (46). Hypocalcemia is prevalent in critically ill patients, and activation of the parathyroid hormone axis along with vitamin D catabolism may ensue as a consequence. Hypocalcemia induces a compensatory elevation in parathyroid hormone and amplifies the conversion of vitamin D 25-hydroxy to 1,25-Dihydroxyvitamin D3. Hypocalcemia stimulates maximal osseous resorption and intestinal calcium absorption to sustain calcium homeostasis (47). Further investigation is necessary to ascertain whether vitamin D supplementation enhances outcome in critically ill patients. Moraes et al. included 135 ICU patients in their study. Mortality rates were significantly higher among patients with vitamin D levels <12 ng/mL compared with those with vitamin D levels >12 ng/mL (32.2% vs. 13.2%), yielding an adjusted relative risk of 2.2 (95% CI, 1.07–4.54; *p* < 0.05). This study suggests that low vitamin D levels at ICU admission are an independent risk factor for mortality in critically ill patients (48). In the meta-analysis conducted by Zhang et al., seven cohort studies involving a total of 4,204 participants, including 1,679 cases of vitamin D deficiency, were analyzed. Vitamin D deficiency was significantly associated with increased hospital mortality (OR, 1.76; 95% CI, 1.38–2.24; *p* < 0.001) (49). de Haan et al. (11) reported that 14 observational studies involving 9,715 critically ill patients were assessed. Concentrations of 25(OH) D below 50 nmol/L were associated with 30-day mortality (RR, 1.42; 95% CI, 1.00–2.02; *p* = 0.05) and in-hospital mortality (RR, 1.79; 95% CI, 1.49–2.16; *p* < 0.001). Previous literature studies have shown that critically ill patients with low vitamin D levels have a higher risk of mortality (11, 48, 49).

### 4.1 Mortality in critically ill adults

Several meta-analyses have shown inconsistent results in recent years. A meta-analysis by Peng et al. (50) revealed no linkage between vitamin D use and reduced all-cause mortality at 30 days, 90 days, and 180 days; all-cause ICU mortality; or all-cause in-hospital mortality. According to Lan et al.’s (51) meta-analysis, there was no significant difference in 28-day mortality between the vitamin D supplementation group and the placebo group (*p* = 0.17). Shen et al.’s (52) meta-analysis revealed that vitamin D administration did not influence the overall mortality compared with the placebo in critically ill adults.

Menger et al. (53) included 16 RCTs involving 2,449 patients in their meta-analysis and reported that vitamin D supplementation significantly decreased overall mortality compared with a placebo (*p* = 0.03). Nine trials reported 28-day mortality, and compared with placebo, vitamin D supplementation tended to reduce 28-day mortality (*p* = 0.06, RR = 0.73). After the VIOLET study was removed, critically ill adults who received vitamin D supplementation experienced a significant reduction in 28-day mortality (*p* = 0.001, RR = 0.68). Kaur et al.’s (54) meta-analysis of 11 RCTs involving 2,328 patients revealed that vitamin D supplementation had no effect on overall mortality compared with placebo or no agent being provided to critically ill patients (*p* = 0.47, OR = 0.93). However, after the VIOLET study was removed, critically ill adults who received vitamin D supplementation experienced a significant reduction in overall mortality (*p* = 0.03, RR = 0.74). Zheng et al.’s (55) meta-analysis of 19 RCTs involving 2,754 participants revealed that vitamin D supplementation significantly decreased the probability of short-term mortality (90-day mortality or hospital mortality) compared with placebo (*p* = 0.03).

More RCTs and patients would have been included would have been included in the meta-analyses, which showed that vitamin D supplementation tended to reduce mortality in critically ill adults. When the VIOLET trial was eliminated in the meta-analysis, vitamin D supplementation was shown to significantly decrease mortality in critically ill adults. The VIOLET trial revealed that the group receiving vitamin D supplementation had a 90-day mortality rate of 23.5%, whereas the placebo group had a mortality rate of 20.6%, corresponding to a difference of 2.9%. In addition, the mortality rate in the vitamin D supplementation group was 17.3% at 28 days, compared with 13.0% in the placebo group, a difference of 4.3%. Numerical analysis revealed that vitamin D supplementation was associated with a harmful trend in critically ill adults in the VIOLET trial (20).

In the meta-analysis conducted by Gao et al. (56) which encompassed 10 RCTs involving a total of 2,058 critically ill patients, the findings indicated that the administration of a single dose of vitamin D3 ranging from 300,000 IU to 540,000 IU did not correlate with a reduction in mortality rates among this demographic. The author postulated that removing the VIOLET research resulted in significant changes in the ultimate outcome of mortality. Several limitations warrant consideration in the VIOLET study, such as the inclusion of patients with mild critical illness and the fact that 23.6% of those in the vitamin D3 cohort exhibited 25-hydroxyvitamin D levels that remained below 30 ng/mL by day 3, all of which are likely to introduce bias that may distort the trial outcomes towards null results. We concluded that vitamin D administration significantly decreased mortality in critically ill adults. Some subgroups of critically ill patients, but not all, can benefit from vitamin D supplementation.

### 4.2 Optimal dosage of vitamin D supplementation for critically ill adults

Clinical results for critically ill patients were not improved by high vitamin D dosages. There might be numerous reasons for this finding. First, vitamin D supplements were offered in an inactive form that required continuous metabolic processes to activate. However, it appears that many critically ill patients are unable to adequately activate native vitamin D (19). Second, a supraphysiological dosage of vitamin D3 was administered, which may have inhibited associated metabolic processes (57–59). However, bolus dosages of 500,000 IU to 540, 000 IU of vitamin D3 should be used carefully since they might increase the risk of fractures, change biochemical indicators, and cause tolerability problems such as gastrointestinal distress (60, 61).

In the subgroup analysis by Gao et al., patients who received 300,000 IU of vitamin D3 had significantly lower 28-day mortality (*p* = 0.003, RR = 0.47) (57). The results of the current meta-analysis revealed that patients who received ≤300,000 IU vitamin D had significantly lower mortality compared with those who received placebo (*p* < 0.0001, RR = 0.56); however, this result was not found in patients who received more than 300,000 IU vitamin D (*p* = 0.76, RR = 0.98). After the VIOLET study was removed, compared with placebo, high-dose vitamin D did not significantly reduce mortality (*p* = 0.12, RR = 0.85). The appropriate vitamin D dosage for acute critical illness is uncertain, and no standard has been developed; further study is necessary to clarify this issue.

### 4.3 Vitamin D administration route for critically ill adults

Following the formation of 25-OH vitamin D in the liver and 1,25- dihydroxy vitamin D in the kidney, vitamin D becomes a hormone that is biologically active. Critical illness may alter the availability of 25-hydroxyvitamin D because of hepatic and renal failure or a reduced level of vitamin D-binding protein (62, 63). The high incidence of gastrointestinal disorders and the unpredictability of enteral absorption in critically ill adults should be considered. Numerous factors might affect the effectiveness of oral supplements. Consequently, there can be considerable delay between the intervention’s delivery and the potential benefits (64, 65).

A subgroup analysis by Shen et al. (52) revealed that parenteral vitamin D treatment was linked to lower mortality. In another subgroup analysis by Gao et al. (56) patients who received vitamin D intramuscularly exhibited a significant reduction in mortality (*p* = 0.003, RR = 0.47). In a meta-analysis by Menger et al. (53), compared with enteral delivery, parenteral administration was linked to lower 28-day and overall mortality. In the current meta-analysis, a significant decrease in mortality was detected in the subgroup of patients whose vitamin D3 was administered by intramuscular injection or intravenous injection (*p* = 0.0006; RR = 0.59). It is unknown whether the gastrointestinal system of critically ill patients can absorb enough inactive vitamin D and whether the liver and kidneys can sufficiently convert it into its biologically active form, given that ICU patients typically have organ malfunction (3, 64). As a result, compared with oral/enteral delivery, parenteral delivery can significantly increase blood vitamin D concentrations. Thus, parenteral vitamin D treatment can be more effective than enteral delivery in terms of reducing mortality.

### 4.4 Effect of baseline 25-hydroxyvitamin D levels on mortality in critically ill adults

A prospective study by Trongtrakul et al. (66) revealed that 30-day mortality was significantly greater in patients with severe sepsis whose baseline 25-hydroxyvitamin D level was less than 12 ng/mL compared with those whose baseline level was greater than 12 ng/mL (*p* = 0.003, OR = 7.69). According to the experiments of Amrein et al. (19) vitamin D3 or placebo was given orally or via nasogastric tube once at a dose of 540,000 IU followed by monthly maintenance doses of 90,000 IU for 5 months and high-dose vitamin D supplementation may be particularly beneficial for the critically ill patients with severe vitamin D insufficiency levels (< 12 ng/mL). In the study by Bhattacharyya et al., patients with sepsis received 540,000 units of vitamin D3 dissolved in 45 mL of milk, administered either orally or via enteral access. The authors performed a subgroup analysis of critically ill sepsis patients with severe vitamin D deficiency whose baseline 25-hydroxyvitamin D level was less than 12 ng/mL and found a tendency for vitamin D supplementation to lower 90-day mortality (*p* = 0.087) (38). The results of the present meta-analysis revealed that mortality tended to decrease in critically ill adults whose baseline 25-hydroxyvitamin D level was less than 20 ng/mL; moreover, critically ill adults whose baseline 25-hydroxyvitamin D level was less than 12 ng/mL experienced a notable improvement in mortality. However, the medical community needs greater understanding in this area and expects that the current international, multicenter VITDALIZE study focused on assessing the effectiveness of high vitamin D dosages in individuals suffering from severe vitamin D insufficiency (serum levels ≤ 12 ng/mL) will explore this critical issue (67). Routine vitamin D supplementation is not recommended for all ICU patients; instead, clinical decisions should consider baseline vitamin D status, dose, and route of administration. Assessing serum 25-hydroxyvitamin D levels in critically ill adults is essential to determine potential benefit. We recommend routine measurement of baseline levels in the ICU prior to supplementation. Vitamin D therapy may be beneficial when baseline serum 25-hydroxyvitamin D levels are <20 ng/mL.

## 5 Limitations

Although this study focused on critically ill adults, the study population was diverse. For example, some patients suffered serious traumatic injuries, while others were taken to the surgical intensive care unit following an operation. Medical ICU patients suffering from sepsis, acute kidney injury, pneumonia, respiratory failure, or shock were included in certain trials. The selected studies varied greatly in terms of sample size. The majority of the trials included in our meta-analysis had small sample sizes, with only three large-scale RCTs (more than 200 patients) included (19, 20, 34). Our meta-analysis employed liquid chromatography–tandem mass spectrometry assay, the reference technique for measuring 25-hydroxyvitamin D levels. But not all of the included studies employed liquid chromatography–tandem mass spectrometry assay to measure the 25-hydroxyvitamin D concentration. The diverse analysis of 25-hydroxyvitamin D data may have introduced bias into the final results. We first evaluated 90-day mortality, followed by 28-day mortality and hospital mortality in included studies. We replaced alternative mortality rates for 90-day mortality, which may have added bias and should be considered with caution. The baseline 25-hydroxyvitamin D level, dosage and route of vitamin D supplementation, and illness severity of the recruited patients differed across the trials. Thus, the variability and evaluated confidence of the studies restrict the generalizability of the conclusions, which should be interpreted cautiously. Subgroup analyses of baseline vitamin D level (less than 20 ng/mL) are based on 16 studies. But subgroup analyses of baseline vitamin D level (less than 12 ng/mL) are based on only two studies, and results should be interpreted with caution.

## 6 Conclusion

This meta-analysis revealed that vitamin D supplementation significantly reduced mortality in critically ill adult patients, but not all patients benefited. The benefit was observed in a subgroup of patients with baseline 25-hydroxyvitamin D levels below 20 ng/mL, those receiving ≤300,000 IU of vitamin D supplementation, and those receiving the supplement via intramuscular or intravenous injection. Measuring baseline plasma 25-hydroxyvitamin D concentration before supplementation should become standard in ICU practice because baseline 25-hydroxyvitamin D levels are important guides for determining whether providing vitamin D supplements to critically ill adults can have any positive consequences. More research is necessary to determine the optimal vitamin D dosage for critically ill adults.

## Data Availability

The original contributions presented in the study are included in the article/supplementary material, further inquiries can be directed to the corresponding author.
